# Small-cell lung carcinoma with ovarian metastasis 4 years after the first-line treatment

**DOI:** 10.1007/s13691-019-00365-7

**Published:** 2019-03-07

**Authors:** Jun Kitazawa, Akimasa Takahashi, Mao Uemura, Yoshihiko Hayashi

**Affiliations:** Department of Obstetrics and Gynecology, Nagahama City Hospital, 313 Oinui-cho, Nagahama, Shiga 526-8580 Japan

**Keywords:** Immunohistochemistry, Laparoscopic surgery, Ovarian diseases, Small-cell lung carcinoma

## Abstract

Small-cell lung carcinoma rarely metastasizes to the ovary. Only few cases of this condition have been reported to date. A 42-year-old female nonsmoker was an outpatient after receiving treatment for small-cell lung carcinoma. Approximately 45 months after the first-line treatment, the pro-gastrin-releasing peptide level exhibited a gradual increase. Positron emission tomography-computed tomography revealed abnormal accumulation in the left ovary. Accordingly, we performed laparoscopic salpingo-oophorectomy. Both pathological and immunohistochemical examinations (thyroid transcription factor-1, synaptophysin, and chromogranin A staining) led to the diagnosis of ovarian metastasis of small-cell lung carcinoma. The pro-gastrin-releasing peptide level declined postoperatively, and no recurrence has been reported thus far. Here we reported an extremely rare case of small-cell lung carcinoma metastatic to the ovary after several years of receiving the initial treatment for small-cell lung carcinoma, which, however, exhibited an excellent course postoperatively.

## Introduction

Small-cell lung carcinoma (SCLC) is a type of lung cancer characterized by high proliferative ability and metastatic potential. Even in patients with SCLC eligible to receive the best treatment, 50% experience recurrence within the first year [1]. SLSC recurrence has been typically reported in organs, such as the brain, liver, lung, and bone. However, its metastasis to the ovary is a sporadic occurrence, and an extensive literature search revealed only few such documented cases. Here, we present a unique case of a patient with SCLC with ovarian metastasis 4 years after receiving the first-line treatment but exhibited good results with surgical treatment.

## Case report

Patient: A 42-year-old female nonsmoker.

BMI: 26.6 kg/m^2^.

Medical history: nothing in particular.

**Fig. 1:**
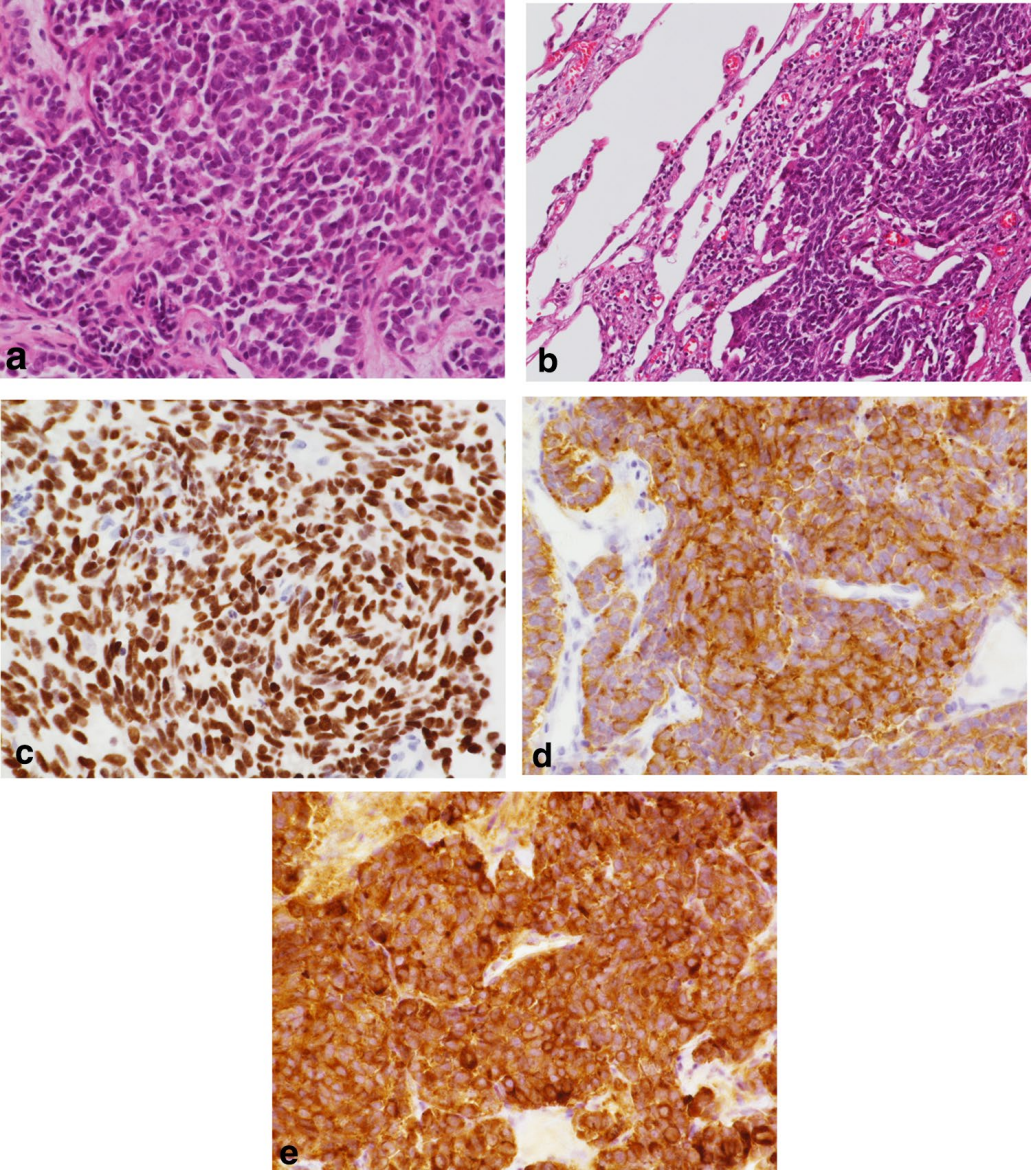
Histological and immunohistochemical findings of the left lower lung lobe. **a** Biopsy tissue: dense growth of heterotypic cells with high N/C ratio is observed. Lesions forming irregular alveolar lamina and nuclear chromatin are relatively uniform; nucleoli are not noticeable (H&E staining, ×40). **b** Surgical sampling: invasive proliferation of heterozygous cells with a high N/C ratio is observed in the region, showing a nodule-like shape microscopically. Nuclear chromatin is in the form of fine granules, and nucleoli are not noticeable (H&E staining, ×20). Immunoreactivity of tumor cells with antibodies to TTF-1 (**c** immunostain, ×40), synaptophysin (**d** immunostain, ×40), and chromogranin A (**e** immunostain, ×40)

Clinical course: She was referred to the Thoracic Surgery Department of Nagahama City Hospital because of an abnormal chest X-ray radiograph (left pulmonary hilar nodule, 3-cm diameter). A bronchoscopic biopsy revealed SCLC (LD: c-T2aN1M0; Stage IIA, Fig. 1a). However, her metastatic workup was negative, which comprised cranial magnetic resonance imaging (MRI) and bone scintigraphy. Accordingly, combined treatment with chemotherapy [60 mg/m^2^ cisplatin (day 1) + 60 mg/m^2^ irinotecan (days 1, 8, and 15)] and radiotherapy (total 36 Gy) was initiated, followed by thoracoscopic lobectomy 3 weeks after last chemotherapy (Fig. 1b). Immunohistochemical findings of lung tissue are shown in Fig. 1c–e. Of note, the surgical stage was yp-T1N0M0: Stage IA.

**Fig. 2:**
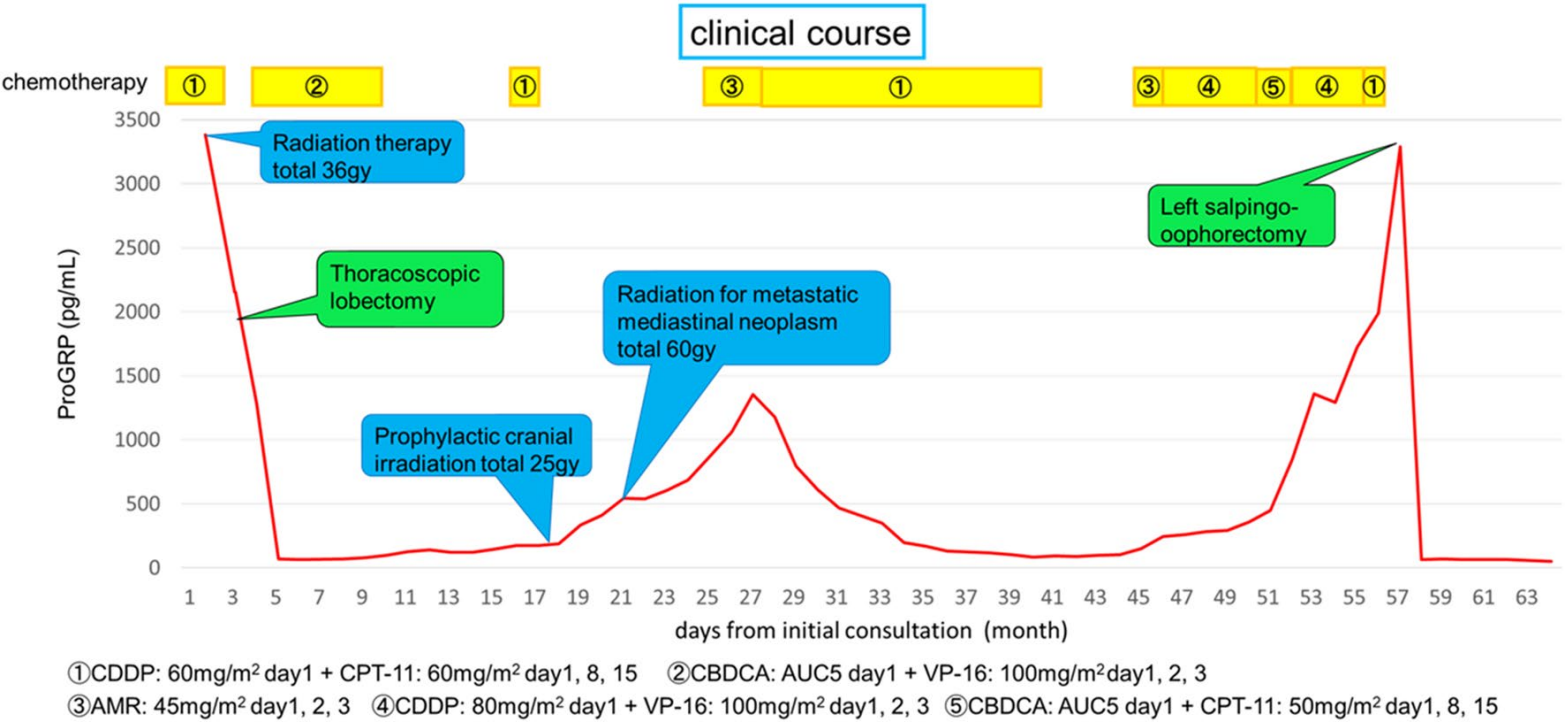
Clinical course from the initial consultation. Approximately 4 years after the first visit, the ProGRP level registered a gradual increase. After surgery, the ProGRP level dramatically declined

Figure 2 shows the postoperative course. Nearly 4 years after receiving the first-line treatment, the pro-gastrin-releasing peptide (ProGRP) level exhibited a gradual increase. The patient underwent additional chemotherapy with amrubicin alone [45 mg/m^2^ (day 1, 2, 3), cisplatin + etoposide [80 mg/m^2^ cisplatin (day1) + 100 mg/m^2^ etoposide (day 1, 2, 3)], and carboplatin + irinotecan [AUC5 carboplatin (day1) + 50 mg/m^2^ irinotecan (day 1, 8, 15)]. Despite this treatment, the ProGRP level did not decrease. Positron emission tomography-computed tomography (PET-CT) revealed an abnormal accumulation in the left ovary. In addition, pelvic MRI revealed a solid tumor in the left ovary with an uneven contrast effect. Accordingly, she was referred to the Obstetrics and Gynecology Department.

**Fig. 3:**
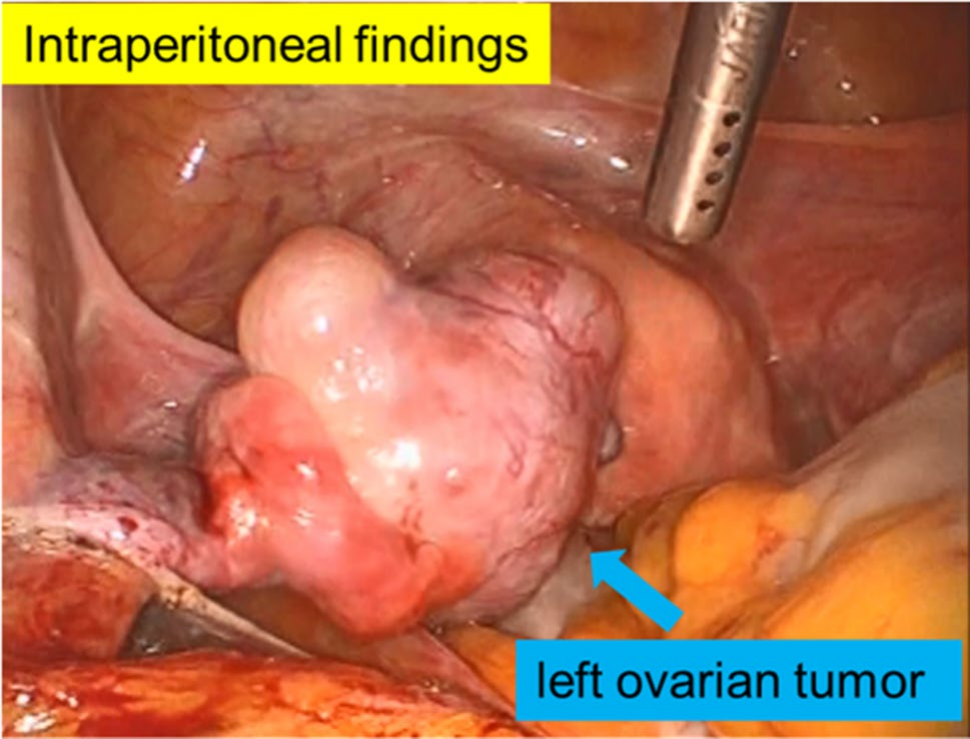
Intraperitoneal findings during surgery. The left ovary was enlarged to 4 cm (blue arrow). The external surface was smooth and formed bumps

**Fig. 4:**
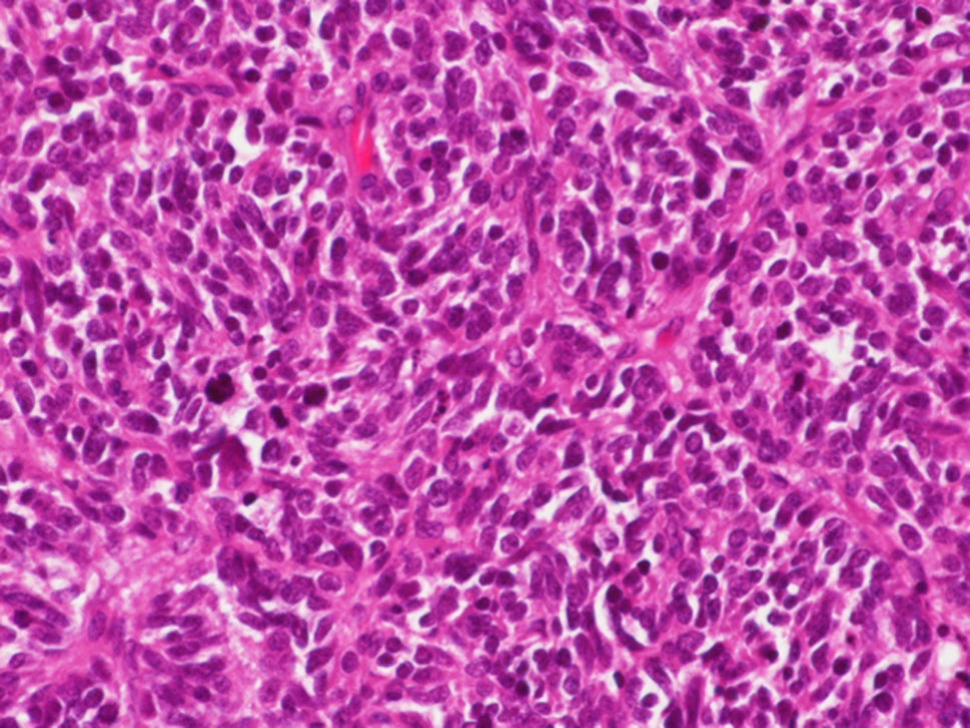
Histological findings of the left ovarian tumor. An irregular alveolar growth pattern of heterotypic cells with a high N:C ratio. Nuclear chromatin was present in fine granular form. The rosette sequence was suspected in part (H&E staining, ×40)

**Fig. 5:**
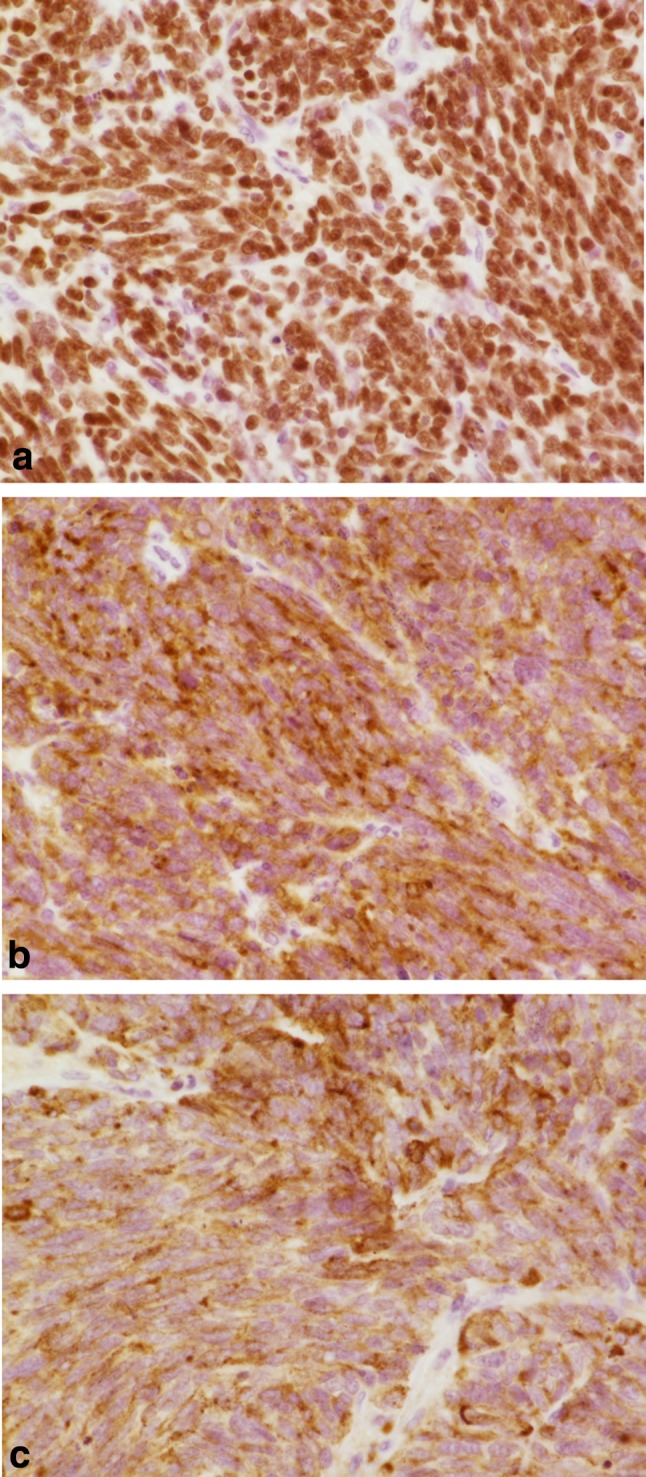
Immunohistochemical findings of the left ovarian tumor. Immunoreactivity of tumor cells with antibody to TTF-1 (**a** immunostain, ×40), synaptophysin (**b** immunostain, ×40), and chromogranin A (**c** immunostain, ×40)

We performed laparoscopic salpingo-oophorectomy 3 days after she visited our hospital. The left ovary had enlarged to 4 cm, and the external surface was smooth and formed bumps (Fig. 3). There was no other metastatic site. Figure 4 shows the pathological histology of the left ovarian tumor. In addition, all immunohistochemistry results for thyroid transcription factor-1 (TTF-1), synaptophysin, and chromogranin A were positive (Fig. 5); these immunohistochemical test results have also been found to be positive in lung tumor tissue (Fig. 1). These findings were consistent with metastatic SCLC in the ovary. Postoperatively, as the ProGRP level markedly decreased, we did not perform any additional treatment. 11 months after this recurrence, her carcinoma has not relapsed.

## Discussion

SCLC is a type of lung cancer that is known to recur in various organs of the body, including the brain, liver, lung, and bone. However, its metastasis to the ovary is a rarely reported event; therefore, only few studies have reported such cases to date. In addition, to the best of our knowledge, no study, to date, has reported a successful case of surgical treatment of ovarian metastasis of SCLC. Thus, this report presents a unique case.

Studies have revealed that SCLC is very aggressive, and metastases to the lymph nodes and distant organs have already been recognized at its diagnosis. However, the frequency of ovarian metastasis from primary lung carcinoma, including SCLC, is very low (0.4%) [2]. Table 1 summarizes cases of ovarian metastasis of SCLC [1–6]. In these cases, ovarian metastasis was detected several months after the diagnosis of SCLC. However, in our case, ovarian metastasis occurred after a prolonged period of 57 months after receiving the first-line treatment. To date, no case of ovarian metastasis this long after SCLC treatment, like our case, has been reported. SCLC is a type of cancer that is synonymous with poor prognosis. The overall 2-year survival is reported to be only 21% in treated patients, whereas untreated patients have a median survival of 3 months since diagnosis [7]. Our patient survived for an exceptionally long period, which might have resulted in ovarian metastasis from SCLC.

**Table 1:** A summary of published reports of SCLC with ovarian metastasis

Author (year)	Age	Time from treatments of lung cancer (month)	Situs of metastases excluding ovaries	Immunohistochemistry
Present case	42	57	Anterior mediastinum	TTF-1, synaptophysin, and chromogranin A positive
Moro F (2017)	24	0	No site	No shown
Garcia V (2010)	54	3	Lymph nodes in the subcarinal, left hilus and left supraclavicular, central nerve system	Synaptophysin, chromogranin A and p53 positive
Irving JA (2005), 14 cases	46 (26–71)	2.2 (0–12)	Various sites (bone, liver, mediastinum etc.)	TTF-1 positive in 4 out of 6 cases
Bing Z (2005)	62	0	No site	TTF-1 and chromogranin A positive
Sukumvanich P (2005)	42	12	Brain	No shown
Malviya VK (1982)	40	0	No site	No shown

Differentiating between ovarian metastasis of SCLC and primary ovarian small-cell carcinoma is challenging as both present similar histological and immunohistochemical findings. Typically, TTF-1, synaptophysin, and chromogranin A tend to test positive for small-cell carcinoma of any organ other than the lung. Although the standard treatment for small-cell lung cancer has been established, a treatment protocol for primary ovarian small-cell carcinoma, which has worse prognosis, has not yet been established. Hence, comprehensive assessment of the whole body to confirm the presence of tumors in other organs, such as lungs, is imperative to differentiate between these conditions.

Regarding pathological differential diagnosis of this case, adult-type granulosa cell tumor, primitive neuroectodermal tumor (PNET), and desmoplastic small round cell tumor can be mentioned. The diagnosis for adult type granulosa cell tumor was considered negative because immunostaining was positive for chromogranin A, synaptophysin, and TTF-1; nuclear furrows could not be confirmed; and many fissile images were observed in the H&E staining. The diagnosis for PNET was considered negative because of H&E staining, poor homogeneity in tumor cells, and nuclear chromatin not being delicate. Also, it was difficult to believe that PNET was rarely generated in ovaries. The desmoplastic small round cell tumor was considered negative because no remarkable interstitial reaction was observed.

In our case, surgical resection of this chemotherapy-resistant ovarian metastasis resulted in a remarkable decrease in tumor marker levels. Usually, recurrent SCLC is treated with chemotherapy, and the antitumor effects of subsequent chemotherapy depend on the period from the initial treatment to recurrence. Some studies have reported that the response rate of chemotherapy is significantly higher in prolonged cases with a period of 60−90 days or longer before recurrence [8, 9]. Johnson et al. reported that regarding the response rate of oral etoposide against recurrent SCLC, the number of patients who relapsed after > 90 days was 64%, whereas those who had < 90 days from the end of initial treatment were 12.5% [9]. In our case, however, nearly 60 days had passed since the last chemotherapy to recurrence, and additional chemotherapy had failed. The findings of our case suggest that despite no benefit of surgical treatment for metastasis of SCLC, it is a curative treatment option if the metastatic lesion is localized.

She was young and a non-smoker, and she did not have any risk factors for SCLC, such as family history or environmental factors. SCLC is rare in non-smokers. In an epidemiologic study performed in the USA, only 2.5% of all SCLC cases were diagnosed among non-smokers [10]. In our case, the reason for the long-term course remains unclear. However, we hypothesize that chemotherapy played a role in delaying the progression of the disease, although it cannot prevent recurrence. On the other hand, it is possible that cancer cells resistant to chemotherapy may have been dormant for a long time. Clinical evidence of cellular dormancy has been documented in both primary tumor and metastases, and it has also been suggested that dormant cells can be refractory to treatment [11]. Because there are few cases on long-term survival of small-cell lung cancer, it is necessary to further accumulate and examine such cases in the future.

In conclusion, we reported an extremely rare case of ovarian metastasis of SCLC after several years of receiving the initial treatment. Successful treatment of this case highlights that surgical treatment could be a potential treatment option for localized metastatic lesions that are resistant to chemotherapy.
